# “Overcrowded but lonely”: exploring mental health and well-being among young prisoners in Cambodia

**DOI:** 10.1108/IJPH-02-2023-0011

**Published:** 2023-06-28

**Authors:** Puthy Pat, Kerstin Edin, Bhoomikumar Jegannathan, Miguel San Sebastian, Linda Richter Sundberg

**Affiliations:** Department of Epidemiology and Global Health, Umeå University, Umeå, Sweden; Center for Child and Adolescent Mental Health (Caritas-CCAMH), Takhmau, Cambodia; Department of Epidemiology and Global Health, Umeå University, Umeå, Sweden

**Keywords:** Young prisoners, Mental health, Cambodia

## Abstract

**Purpose:**

Young prisoners are one of the most vulnerable groups in society for mental health problems and ill-being. Therefore, there is a crucial need to understand their physical, psychological and social situations. This study aims to explore young Cambodian prisoners’ experiences and perceptions of mental health and well-being, their determinants and their coping strategies.

**Design/methodology/approach:**

Six focus group discussions were carried out in three prisons with a total of 48 young prisoners between the ages of 15 and 24 years (50% women, 50% men). Semi-structured questions guided the discussions, and thematic analysis was applied to analyse the data.

**Findings:**

Young prisoners reported multifaceted experiences of mental health and well-being. The majority described adverse mental health experiences, while some revealed better well-being, partly influenced by the socio-economic support from outside the prisons and previous involvement or not in drug abuse. The experience of physical overcrowding without emotional attachment among the fellow prisoners was perceived as the overarching determinant of loneliness and mental health problems, while socio-emotional support and rituals were described as the most important coping mechanisms.

**Originality/value:**

This pioneering study from Cambodia gives young prisoners an opportunity to voice their experiences and perceptions of mental health and well-being in the prison setting. The findings in this study underline the importance of prison authorities tackling overcrowding to promote well-being and reduce mental health problems. Also, the coping mechanisms outlined by the participants should be considered when planning psychosocial interventions.

## Background

The World Health Organisation (WHO) defines mental health as “a state of mental well-being that enables people to cope with the stresses of life, realize their abilities, learn well and work well, and contribute to their community” (WHO, 2022). Prisoners are considered to be at high risk for mental and physical health problems compared to the general population (Dirkzwager *et al.*, 2021; Butler *et al.*, 2022). This has been reported globally, including studies from both high-income countries (HICs) (Kurdyak *et al.*, 2022) and low- and middle-income countries (LMICs) (Reta *et al.*, 2020). A rapid review on the prevalence of mental health showed that mental disorders among prisoners in LMICs were higher compared to HICs (Hill *et al.*, 2022). In addition, a systematic review from LMICs reported a 16 times higher risk of mental disorders among prisoners compared to the general population (Baranyi *et al.*, 2019).

Well-being is a positive state which encompasses the interconnection of several aspects, including overall health, optimum nutrition, connectedness in the society, safe and supportive environment, knowledge, competence and resiliency (Ross *et al.*, 2020). Young prisoners, in particular, are more vulnerable to impaired well-being and mental health problems compared to older prisoners (Gonçalves *et al.*, 2016). In comparison to youth in the general population, young prisoners have reported three times higher rates of mental disorders (Heller *et al.*, 2021). This vulnerability has been linked to both pre-incarceration and incarceration factors. The former includes childhood trauma, including sexual abuse (Liu *et al.*, 2021), together with many other different adverse childhood experiences, such as poverty, unemployment, homelessness, substance abuse, lack of education and previous health problems (Kurdyak *et al.*, 2022; Turner *et al.*, 2021).

The incarceration process, at all phases, may also have an impact on the mental health and well-being of the prisoners. Once entering the prison, the environment and psychosocial setting cause anxiety due to the loss of autonomy and adjustment problems that may also lead to somatic problems (Alves *et al.*, 2016). While in the prisons, young people are often vulnerable to interpersonal problems and trauma due to bullying, violence, abuse, isolation, limited meaningful activities, lack of privacy, absence of social networks and lack of future prospects (Durcan and Zwemstra, 2014), all of which are risk factors for mental health problems and ill-being (Abdu *et al.*, 2018; Jordaan *et al.*, 2018). Additionally, studies have shown that overcrowding in prisons is a key factor influencing the well-being and mental health of young prisoners (MacDonald, 2018). A qualitative study among detained Filipino adolescents found that mental health was one of the commonly reported eight key health issues, while food, overcrowding and hygiene were mentioned as the main determinants of their health (Blount, 2020).

From a public health perspective, it is important to understand young prisoners´ mental health and well-being and to identify what kind of support they need to improve their current and future situations (Ruiz‐Íñiguez *et al.*, 2021). Without a supportive environment and access to operating quality services, deterioration of the mental health and well-being of young prisoners might have substantial negative effects on their social and physical situation in the prisons (Chappell and Maggard, 2021). In addition, these negative impacts may continue into their adulthood and significantly affect their well-being, including their education and career (Seker *et al.*, 2022). Young prisoners with mental health problems have also been shown to be at higher risk for violence and recidivism (Barra *et al.*, 2022).

Research on the mental health and well-being of young prisoners in LMICs is scarce, particularly in the Southeast Asian region (Welu *et al.*, 2021). While the mental health of young prisoners in Cambodia has previously been studied using quantitative methods (Pat *et al.*, 2021), no study has captured the voice of this population regarding how they experience their mental health and well-being, influencing factors, and the mechanisms used to handle and address the situation during imprisonment. This study aimed to explore young prisoners’ experiences and perceptions of mental health and well-being, their perceived determinants, as well as their coping strategies in the Cambodian prison setting.

## Method

### Setting

Cambodia suffered from more than three decades of civil war with a traumatic history. During the Pol-Pot era at the end of the 1970s, around two out of seven million people died due to execution, torture, landmines, overwork or starvation (Grundy *et al.*, 2009). The health system was completely destroyed but has been gradually revitalised since 1979 (Grundy *et al.*, 2009; Clarke *et al.*, 2016).

There are 24 prisons spread over the country, one in each province, with a total of 5,552 young prisoners (347 women and 5,205 men) between 15 and 24 years of age (GDP, 2016). There are separate buildings for female and male prisoners, often gardens for prisoners to grow vegetables, rooms for spiritual practice and a library. Overcrowding is a major issue in Cambodian prisons; for example, around 50 prisoners can be living in a room of about 50 m^2^. In each room, a senior prisoner is selected by the prison management team to act as a “leader” for the group in that room. To the best of our knowledge, all prisoners with different severity levels of crimes and/or convictions live together in the same spaces, and there is no solitary confinement in the Cambodian prison system. There is generally one health post in each prison with nurses and/or auxiliary nurses who provide basic health care and refer prisoners with severe physical or mental health problems to the nearest hospital.

### Selection of participants

Three geographically representative prisons were selected: one each from the north, the centre and the south of Cambodia. Young male and female prisoners aged 15–24 years who were under appeal and convicted were eligible for the study. Those prisoners who were accused and arrested but not yet convicted and those who were residing in the prisons for less than six months were not eligible for this study. The prison authorities provided a list of 739 eligible young prisoners, and the research team randomly selected 48 of them (24 men and 24 women). They were invited to focus group discussions (FGDs), and all of them agreed to participate. Around one third (27%) were aged 15–19 years old, while about two thirds (73%) were 20–24 years old. Most of the participants (77%) were not married.

### Procedure

FGD is a type of interview method commonly used in qualitative research to gather the views of participants with experiences around a specific topic (Braun and Clarke, 2006). FGD was selected as the most suitable data collection method for this study, as it gave opportunities for the young prisoners to interact with each other in the group, as well as to explore both individual and group-level experiences and perceptions of being incarcerated and of their physical and mental health challenges. In addition, as prisons are restricted places and because discussing mental health in the prison setting might be sensitive, FGDs are, compared to in-depth interviews, an appropriate approach to reduce the risk of being identified and/or pointed out. Six FGDs were conducted, two in each of the three prisons – one group each for women and men, exclusively. The first FGD was held in November 2018, and the rest were carried out between February and September 2019. The FGDs were conducted in Khmer, the participants’ native language. The prison authorities organised a small room in the library or health post with the intention to give privacy and to minimise disturbances. The session lasted approximately 1 hour per group, and the proceedings were audio recorded after receiving informed consent from the participants and prison authorities. Prison authorities expressed the need to be present during the FGDs for safety reasons. However, the research team felt the participants might be constrained in their responses in the presence of prison authorities and therefore requested the authorities to be seated far away to avoid grasping the content of the discussion. The authorities complied with our request, and the interviews were conducted without their direct observation, which we believe gave more privacy and confidence for the young prisoners to openly air their concerns and opinions during the FGDs.

Using a semi-structured approach, a guide with open-ended questions was prepared by the research team to achieve the objectives of the study. The FGDs explored the participants’ experiences and perceptions of mental health and well-being, followed by discussions about perceived determinants, possible coping strategies and access to support and care if needed. During the FGDs, participants expressed their own experiences and perceptions of mental health and well-being as well as observations of other young fellow prisoners.

### Analysis process

Thematic analysis was used to analyse the data following the guidelines from Braun and Clarke (Braun and Clarke, 2006) using an inductive approach. The first author, who is Cambodian and a native language speaker, manually transcribed the audio recordings and read the transcripts several times to cross-check the authenticity before handing them over for professional translation to English. The data were analysed line-by-line to generate initial codes with a constant cross-check between the transcripts of English and Khmer versions and the memos. Throughout the whole analytic process, a constant comparison was applied by going back and forth to review all the steps in discussions with the group of researchers. The initial codes were grouped into potential sub-themes and named provisionally (Table 1). The sub-themes were sorted into groups with similar contents and directions to create a set of new potential themes. To ensure that they accurately captured the meaning and content of the FGDs, the potential themes were refined and renamed several times to generate the final themes (Table 1).

### Ethical clearance

The National Ethics Committee for Health Research, Ministry of Health, Royal Government of Cambodia, granted ethical clearance for this study (ref no. N33NGCHR), and the permission to have FGDs in the prisons was approved by the General Department of Prisons, Ministry of Interior, Royal Government of Cambodia. Oral and written consent was sought from each participating prisoner. The facilitator strongly emphasised the confidentiality, safe storage of data, anonymity in report/publication, voluntariness and options to withdraw from the study at any time with no negative consequences.

## Results

**Figure 1:**
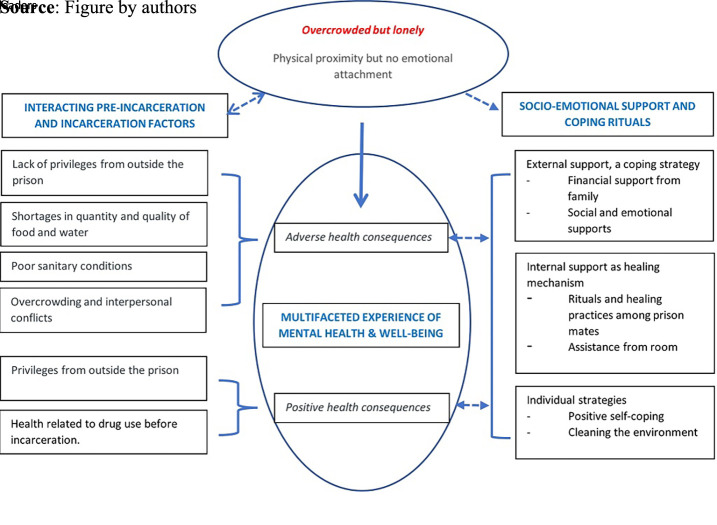
Mental health and well-being of young prisoners, depicting the complex relationships between social determinants and incarceration factors

The outcomes of the thematic analysis are presented as three themes (Figure 1):

1. multifaceted experiences of mental health and well-being;
2. interacting pre-incarceration and incarceration factors; and
3. socio-emotional support and rituals as coping mechanisms.

Overcrowding was an overarching issue that came up across all three themes. The participants often felt lonely among the multitude of fellow prisoners and felt psychologically and emotionally detached from the others despite the physical nearness in the overcrowded setting. The ‘overcrowded but lonely’ experience among the participants affected their health (theme 1), influenced by different determinants of health (theme 2), prompting several coping mechanisms like traditional practices (theme 3).

## Multifaceted experiences of mental health and well-being

The young prisoners described their current health situations in various ways, ranging from very poor to fair or even good.

### Adverse health consequences

In all FGDs, participants reflected that, despite living in overcrowded prisons, they often experienced feelings of loneliness. They were physically connected with each other but mentally and emotionally disconnected, and they felt lonely because they were separated from their loved ones:

Living here, I have no relatives, good friends, or people. I can’t talk about my difficulties, and I feel so lonely. [Another person said] There are too many people, and I have no place to sleep or walk around, but I am alone here. They are different from me, and I can’t talk about my problems […]. (FGD 3, men)

The participating young prisoners expressed several symptoms that they interpreted as indicators of overall poor health. Symptoms mentioned as commonly experienced among men were headache, skin and stomach problems, flu symptoms, coughing, vomiting, dizziness, weakness and numbness, while women often mentioned reproductive health concerns, such as irregular menstrual cycles:

The health is not good, my energy is low, and I am tired, have stomach problems […] and scabies. It is very uncomfortable and difficult to live […] I am feeling nausea and keep losing weight. (FGD 1, man)

[…] different between living outside and in here… my period is irregular […] after I moved here, sometimes it comes once in two/three months. (FGD 2, woman)

Further, most participants had experienced mental health problems at some point. The severity of the mental health problems differed between individuals, from facing low energy to having suicidal thoughts. They described negative emotions and thoughts such as guilt and self-blame, particularly during the first phase of incarceration. They regretted their illegal actions and were concerned about the consequences for their families. For some, these thoughts became intrusive and disturbing, worsening their feelings of worry and shame. Further, this negative cycle increased their stress, and as a result, they faced sleep disturbances and other physical problems, such as headache or body pain:

[…] all of us admitted that we committed a big mistake and that cannot be forgotten or forgiven. My family suffered from it […] we think about our mistakes too much, and I can’t stop it. I can’t sleep, and my health is poor […]. (FGD 4, woman)

Further, women seemed to express more internalising problems such as depressed mood, hopelessness, sadness, worry and anxiety that they found very hard to deal with. Overthinking and thoughts on useless things, a form of rumination, were often mentioned:

All the time, I keep thinking of everything useless, especially thinking about bad things. It come automatically and all the time. I can do nothing about it, but I can’t stop it. I can’t sleep because of such worries. (FGD 4, woman)

Sometimes, these negative thoughts about the future led to isolation and suicidal ideations and behaviour. Additionally, fellow prisoners’ suicidal behaviour caused distress and feelings of helplessness in co-prisoners, as expressed by one woman:

I saw a person in my room who was thinking too much, and she could not stop her useless thinking. She worried about her future […] she wanted to die […] tried to hang herself. (FGD 2, woman)

### Positive health consequences

The majority of participants described many health challenges, but some reported better health in prison. Those who had been exposed to poverty and drug use prior to imprisonment felt an overall improvement, since they had access to more food and no access to drugs:

Enough food, place and time to sleep […] gained weight […] our health is better […], I didn’t eat enough because my family is poor […]. (FGD 1, man)

Women prisoners believed that they were healthier compared to men because they more often shared items and had certain privileges in the prison setting. They showed solidarity with each other, provided emotional and psychosocial support and shared food and items like sanitary products, beddings and cosmetics. They mentioned that the officials were friendlier and kinder to them compared to the male prisoners:

I think that women are doing better than men […] a lot of people living here, and it is crowded. Women share food with each other. (FGD 6, woman)

## Interacting pre-incarceration and incarceration factors in experiencing health

The participants described how incarceration and pre-incarceration factors interacted while they were imprisoned and perceived them as determinants of their mental health and well-being.

### Overcrowding and interpersonal conflicts

Overcrowding led the young prisoners to overthinking and rumination, with some participants expressing feelings of loneliness despite the physical proximity of other mates. Additionally, the lack of space provoked arguments, irritability, and further conflicts and violence, sometimes leading to injuries. Interpersonal relationships among the prisoners were aptly described by a Khmer proverb, “Dishes in the Basket” (“ចានក្នុងរាវ” in Khmer), which means when there are many dishes in the same basket, there will be a crash and one or more will be broken. The metaphor referred to how the overcrowding in the rooms easily led to interpersonal conflicts, loneliness and mental health problems:

When we first stepped in here, […] we felt it’s too crowded. At home, we lived quietly. We now live alone […] used to live with the family of five to six members. It makes us feel lonely and miss our homes […]. (FGD 5, man)

Participants further linked overcrowding to physical and mental health problems. Women tended to refer to this situation of overcrowding in terms of its effects on their bodies, such as decreased weight and changed appearance, as their face looked more wrinkled, while men mainly described overcrowding as a source of interpersonal tensions. Some prisoners were mentally affected by the conflicts and fighting; they became sleepless, helpless, fearful and worried about their safety:

There are a lot of people in the room, sitting or lying down […] I stepped on his feet unintentionally; he scolds me. Then I think a lot […] It leads to sleep disturbance and feeling sad. (FGD 3, man)

### Shortages in quantity and quality of food and water

The participants expressed how they struggled to fulfil their basic needs, such as water supply and food, which affected their health. Though they were provided two meals per day, most of them complained about the lack of access to enough nutritious food. They described how rice and vegetables could have mould and the water was unclean, which led to physical and mental health problems:

Soup is tasteless; the rice is saltless and watery. Sometimes, it is uncooked or overcooked or burnt […] just seeing that rice I am feeling nauseous. We become weaker and tired, and it’s difficult to live. We feel sad […] What can we do? This is prison. (FGD 1, man)

### Poor sanitary conditions

The environmental conditions of the prisons were discussed by participants as one of the key determinants of poor health. They stated that the diverse socio-economic and educational backgrounds were related to different levels of prior knowledge on hygiene and sanitation, which contributed to an uncomfortable ambience of the rooms and other common places in the prisons:

There is not enough air in the room, and the hygiene is not good. We can be contaminated if we stay near the ones who have poor hygiene. While sleeping we need to wear socks, otherwise we may become sick. (FGD 2, woman)

### The (lack of) privileges from outside the prison

The young prisoners’ different health situations in the prison were to some degree also influenced by their socio-economic conditions before incarceration. Those who had financial resources or strong social networks outside the prison also had privileges in the prison, such as better access to food and/or space:

[…] some families that are rich offer things to their relatives here every month. For the poor, no one visits them for months or even years. Therefore, these people have no food, money […] their health is also poor. (FGD 3, man)

### Health related to drug use before incarceration

The issues of drug use affected both users and non-users prior to incarceration. Drug users´ health improved in the prisons:

[…] when they come here, their health is stronger […] it was worse when they were outside because they used illicit drugs […]. (FGD 5, man)

I was thin when outside because I used illicit drugs. Since I came here, I have never used drugs, so I gain weight […]. (FGD 2, woman)

Prior drug-free individuals, however, felt that the loss of freedom made their health become worse:

I don’t use drugs […] My life was much better, as I ate enough and slept well, but when I am here, I have difficulties with everything. (FGD 5, man)

## Socio-emotional support and rituals as coping mechanisms

Young prisoners were often able to practise coping strategies to alleviate their mental and physical health challenges despite the overcrowded environment. The participants sought support and applied strategies from both inside and outside the prison setting.

### External support contributing to health and well-being

The participants described that one of the crucial factors that contributed to the improvement of their mental health and well-being was to receive support from outside the prisons, particularly from family members or extended relatives. The external support could come in different forms, such as emotional support, financial aid and legal assistance:

[…] some families sent money or food to prisoners here, and they look healthier, but who are not [those not receiving financial support from outside] always get sick or upset […] (FGD 6, woman)

### Internal support and traditional healing

The participants emphasised how support from their prison-mates, room leaders and recreational activities facilitated their life in prison. This help was expressed in different ways, from physical care such as massage and coining to verbal comfort. Coining is a traditional healing method in Cambodia in which deep pressure is applied to the skin of the back or chest using coins or other smooth-edged objects:

If the health problem is not so serious, we will manage the problem in the room by coining, giving medicines, and massaging each other […]. (FGD 1, man)

In case of mental health problems, the young prisoners normally assisted each other by sharing their difficulties and comforting one another:

There is a room leader who controls everything here. She is very nice and kind to us. My room leader is a person who could understand others’ feelings or difficulties. She knows the needs of the young people like us. They play good music for us in the evening. (FGD 6, woman)

The participants described how they engaged themselves in various activities organised in the prisons. Prisoners who normally slept next to each other or shared common interests became friends. Usually, they invited each other to play sports, do exercises, watch TV and/or have chats, which, at least for a short time, helped them to forget all the sadness and worries:

We do something happy to let ourselves enjoy the time here. We have a chit chat with our friends, read books, watch movies, and, especially, we sing songs together and play some simple games. This can distract our depressed mood from day to day. (FGD 4, woman)

### Self-help strategies

Prisoners mentioned that they continuously tried to remind themselves to be a strong person to deal with and adapt to the restrictions imposed on them during incarceration. This attitude helped them to distract themselves from sadness and/or irrational thinking about themselves. Some of them had tried to force themselves to think about positive matters, but it was not always possible to do so:

We all have those mental issues, but some of us know how to control those issues by spending time doing good things and thinking positively about ourselves. This is very important for all of us. Sometimes, others do not realise that we are depressed, so we have to help ourselves. (FGD 5, man)

Another strategy to live healthier, both physically and mentally, was to have hygiene routines, such as cleaning oneself as well as the immediate environment. There was an emphasis on taking responsibilities by maintaining hygiene in certain places, such as the eating and sleeping spaces, as per the routine in the prisons:

[…] if everyone wants to be healthy, we should clean our place and stay hygienic, and keep the food waste in the dustbin. If only one person is clean and others are not, it doesn’t work. (FGD 6, woman)

## Discussion

The young prisoners voiced their experiences of mental health and well-being in the context of overcrowding, other perceived determinants and their coping mechanisms. The multifaceted experience of being in the prisons (theme 1) and the pre-incarceration and incarceration factors determine mental health and well-being (theme 2). The socio-emotional support and collective and individual strategies practised by the young prisoners helped them to overcome the adverse health consequences (theme 3).

Our study revealed a variety of complex mental health and well-being challenges faced by young prisoners, which are in line with other studies. For example, a study on infant–juvenile mental health services in Cuban prisons reported that feelings of guilt, tension and frustration were common among the participants (Ruiz‐Íñiguez *et al.*, 2021). Another study with inmates in Colorado revealed that stress, fear, anxiety and other psychiatric symptoms were frequently experienced in this population (Binswanger *et al.*, 2011).

The experience of loneliness despite the overcrowded prison environment was an important finding in our study. Similar results have been reported in England and Wales and Norway, where prisoners often experienced a specific form of loneliness due to the psychological burden of imprisonment and the oppressiveness caused by crowding (Schliehe *et al.*, 2022). In addition, prisoners with a feeling of loneliness were found to have lower levels of self-esteem (Coticchia and Putnam, 2021), increased behavioural and health problems and maladaptive ways of coping with stress (Jozan, 2020). Another study on the relationship between loneliness and psychological effects in both adult and juvenile prisoners in China found that loneliness due to a lack of social support in the prison was directly and significantly associated with poor mental health status (Zhao and Shi, 2020). This explains why participants in our study connected the feeling of loneliness with the experiences of mental health problems and lack of well-being.

In our study, we also found that while most of the participants experienced mental health problems, some experienced improvements, particularly drug users before imprisonment. This finding has been corroborated by a qualitative study that reported incarcerated youths felt safe in the structured environment, experiencing respite from their chaotic lives outside the prison (Barnert *et al.*, 2015). On the other hand, Alves *et al.* (2016) reported that withdrawal symptoms due to alcohol and drug addictions were a challenge, as there was no support for such severe problems in the prisons. Also, a study on the correlation between mental health and substance abuse in the USA found that juveniles who used illegal drugs prior to incarceration reported significantly higher rates of mental health problems compared to their counterparts (Proctor and Kopak, 2021). Another study on young offenders’ perspectives on alcohol and drug health prevention programmes highlighted the importance of educating young prisoners to abstain from drug abuse to promote mental health and well-being (Deans *et al.*, 2022), which reflects our study population, the majority of whom are in prison due to drug abuse-related crimes.

In our study, female prisoners, compared to males, perceived better mental health. They attributed this to either solidarity between themselves as women or to the attention and care from the prison officials. This has also been reported in a study from Indian prisons which found that women were provided more services compared to men in terms of parenting and counselling classes, support groups and day care services, contributing to significantly reduced mental health challenges (Mishra *et al.*, 2021). In contrast, a study on the mental health of youth detainees in the USA found that male juveniles experienced more fairness in care than their female counterparts, which was found to have significant positive effects on their internalising and externalising problems (Sichel *et al.*, 2022). This differential perception of prison settings among men and women may indicate gender issues in different contexts and cultures within the prison system of care and available privileges in high- and low-income countries that need further exploration.

The identified social determinants of health, such as lack of family support and visits, overcrowding, hygienic problems and shortages in quality and quantity of food, were linked by the participants in our study to poor mental health and lack of well-being. Studies conducted in the USA have found that a lack of support from extended families contributed to deterioration of the mental health of young prisoners (Richardson and Brakle, 2011), whereas in Chile, those who had supportive family relationships reported better mental health (Gabrysch *et al.*, 2020). A study from Zambia reported that family wealth, overcrowding, sanitation and nutrition were linked to mental health problems among prisoners (Topp *et al.*, 2016). In addition, a review on the effects of family visits on prisoners’ well-being found evidence of reduced depressive symptoms among young prisoners (De Claire and Dixon, 2017).

Internal support from prison-mates and room leaders and traditional healing practices such as coining played essential roles in reducing health and mental health problems in our study. This is in line with the findings of a mixed-method systematic review where peer support (South *et al.*, 2016) and traditional healing practices in LMICs (Hyatt, 2013) were found to be crucial to improve prisoners’ health. The mutual support among the prisoners was further emphasised by a study from Poland that reported supporting each other was the main source of resilience, life satisfaction and mental well-being among prison youths (Konaszewski *et al.*, 2021). A study in the UK reported similar findings in which positive peer interactions were strongly associated with better well-being and fulfilment of psychological needs (Kyprianides and Easterbrook, 2020), underscoring the importance of peer support system in prisons.

## Methodological considerations

Several measures were taken to enhance the trustworthiness of the study. The authorities complied with our request to be seated away from the discussion, which maximised the level of openness of the participants. Emphasising the voluntary nature of participation, confidentiality of participants’ opinions and freedom to express their experience in the group created a trustful environment, thereby enhancing the credibility. However, the use of FGDs might have reduced some aspects of credibility since confidentiality cannot be fully guaranteed in the group interaction, and this might have hindered the participants from sharing certain personal issues related to their mental health and well-being. Though only three prisons were selected, we still believe that they are representative of other prisons in the country, as they are similar in terms of conditions, rules and regulations, infrastructure and type of prisoners, which supports the transferability of the findings. However, as our participants were in the age range of 15–24 years, the findings might not be generalised to other age groups.

## Conclusion

To the best of our knowledge, this is a pioneering study exploring the experiences and perceptions of young prisoners’ mental health and well-being in Cambodia, a LMIC and post-conflict setting. Loneliness in the overcrowded prisons was an overarching concern, indicating the need for prison reforms to provide more space. Interventions such as ensuring peer solidarity, training room leaders to be mental health first-aid providers, promoting culturally accepted non-harmful traditional practices and ensuring family visits may be considered to address the mental health needs of young prisoners.

## Figures and Tables

**Table 1:** Example of the analysis process

Transcripts	Initial codes	Sub-themes	Theme
*…some families give money to their relatives here…*	*Family financial support*	*External support plays an essential role*	*Socio-emotional support and rituals as coping mechanisms*
*…we have relatives visit us and talk about good things…*	*Family emotional support*		
*manage the problem in the room by coining…*	*Coining as healing ritual*	*Recreational, ritual activities*	
*…I like to watch TV … and play ball with others …*	*Watching TV, playing sports*		
*… room leaders play good music for us in the evening …*	*Emotional comfort*	*Supportive role of the room leaders*	
*…room leaders inform officials when we are sick…*	*Informing officials*		

**Source:** Table by authors
